# Density functional theory investigation of the contributions of π-π stacking and hydrogen bonding with water to the supramolecular aggregation interactions of model asphaltene heterocyclic compounds

**DOI:** 10.1007/s00894-024-05922-3

**Published:** 2024-04-24

**Authors:** Milena D. Lessa, Stanislav R. Stoyanov, José Walkimar M. de Carneiro, Leonardo M. da Costa

**Affiliations:** 1 Programa de Pós-Graduação Em Química, Departamento de Química Inorgânica e Departamento de Química Orgânica, Instituto de Química, Universidade Federal Fluminense, Outeiro de São João Batista S/N, Niterói, RJ 24020-141 Brazil; 2 Natural Resources Canada, CanmetENERGY Devon, 1 Oil Patch Drive, Devon, AB T9G 1A8 Canada

**Keywords:** Heterocycles dimers, DFT, Interaction analysis, Chemical scaling

## Abstract

**Context:**

A complex supramolecular process involving electrostatic and dispersion interactions and asphaltene aggregation is associated with detrimental petroleum deposition and scaling that pose challenges to petroleum recovery, transportation, and upgrading. The homodimers of seven heterocyclic model compounds, representative of moieties commonly found in asphaltene structures, were studied: pyridine, thiophene, furan, isoquinoline, pyrazine, thiazole, and 1,3-oxazole. The contributions of hydrogen bonding involving water bridges spanning between dimers and π-π stacking to the total interaction energy were calculated and analyzed. The distance between the planes of the aromatic rings is correlated with the π-π stacking interaction strength. All the dimerization reactions were exothermic, although not spontaneous. This was mostly modulated by the strength of the hydrogen bond of the water bridge and the π-π stacking interaction. Dimers bridged by two water molecules were more stable than those with additional water molecules or without any water molecule in the bridge. Energy decomposition analysis showed that the electrostatic and polarization components were the main stabilizing terms for the hydrogen bond interaction in the bridge, contributing at least 80% of the interaction energy in all dimers. The non-covalent interaction analysis confirmed the molecular sites that had the strongest (hydrogen bond) and weak (π-π stacking) attractive interactions. They were concentrated in the water bridge and in the plane between the aromatic rings, respectively.

**Methods:**

The density functional ωB97X-D with a dispersion correction and the Def2-SVP basis set were employed to investigate supramolecular aggregates incorporating heterocycles dimers with 0, 1, 2, and 3 water molecules forming a stabilizing bridge connecting the monomers. The non-covalent interactions were analyzed using the NCIplot software and plotted as isosurface maps using Visual Molecular Dynamics.

**Supplementary Information:**

The online version contains supplementary material available at 10.1007/s00894-024-05922-3.

## Introduction

In the last decades, the efficient extraction and transport, marine and terrestrial, of crude oil have become an increasing challenge for the petroleum industry, mainly due to organic and inorganic depositions [1, 2]. The formation of insoluble aggregates occurs on the surfaces of practically all oil production and transportation systems: in pipelines, reservoirs, containment screens, submerged or surface installations that significantly reduce the recovery of oil [3, 4]. This generates a large financial loss for the oil industry that, as preventive or remedial measures, must stop production for cleaning or maintenance to increase the useful life of the equipment and the continuity of oil recovery [4–6].

Asphaltenes, the densest and most polar fraction of petroleum, are considered the main generators of organic deposition in oil production and transportation systems [7, 8]. They are a mixture of large molecular weight organic molecules containing polyaromatic chains, side alkyl groups, polar functional groups, and heterocycles with O, N, and S as heteroatoms [9, 10]. Regarding the molecular structure, asphaltenes can be considered belonging to the continental model, containing a central aromatic moiety with more than seven fused rings and pendant aliphatic chains, or the archipelago model, formed by smaller aromatic moieties interconnected by bridges of alkyl groups [10, 11]. Experimental and computational studies show that these structures exhibit a strong aggregation preference in solution, forming insoluble solids that reduce oil recovery [12–20]. Asphaltene aggregation is also detrimental in the context of spill response in the event of accidental oil spills in water [21, 22].

**Fig. 1:**
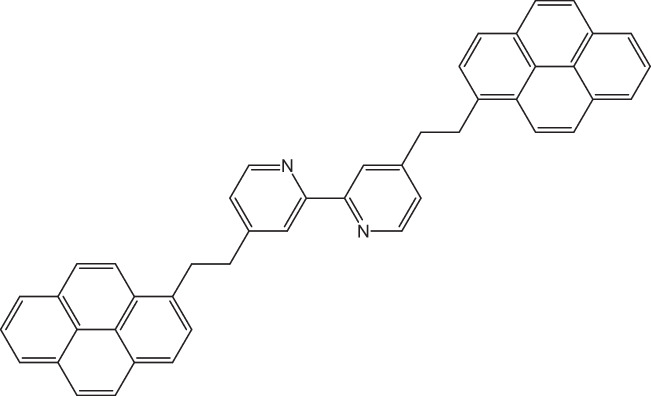
Model structure of 4,4′-bis(2-pyren-1-yl-ethyl)-2,2′-bipyridine (PBP)

Due to the complex constitution of asphaltenes, the insoluble aggregates are stabilized by dispersive, polarization, and electrostatic interactions, arising mainly from London forces, π-π stacking, exchange-repulsion contributions, hydrogen bonding, and dipole–dipole interactions [23–26]. Recently, Hassanzadeh and Abdouss [27], based on studies using the supramolecular assembly model by Gray et al. [28] and the nanoaggregate model by Yen–Mullins [29], have proposed a supramolecular organization model of asphaltene aggregation that combines cooperative binding by acid–base interactions, hydrogen bonding, π-π stacking, and metal coordination, as well as the formation of hydrophobic pockets, porous networks, and host–guest complexes. All these diverse supramolecular interactions are important for the attraction between asphaltene molecules that leads up to generate organic scaling. Based on the supramolecular assembly model [28], Gray et al. have developed organic molecules that would experimentally simulate the aggregation behavior of asphaltenes [30]. Figure 1 shows the structure of one of these synthetic asphaltene molecules that contain a bipyridine tethered with ethyl groups to two pyrene moieties.

In previous works [31, 32], we investigated computationally the interaction between model asphaltene compounds, reported by Gray et al., to rationalize the experimental information that water traces could enhance the aggregation behavior of asphaltenes. We showed that water molecules could interact with the N atoms of the stacked dimers of asphaltene model compounds, such as PBP and its analogs with varied aromatic hydrocarbon groups. The interaction strength comparison between the π-π stacking and hydrogen bond showed that these interactions have almost the same contribution to the stabilization of the nanoaggregate [31, 32].

**Fig. 2:**
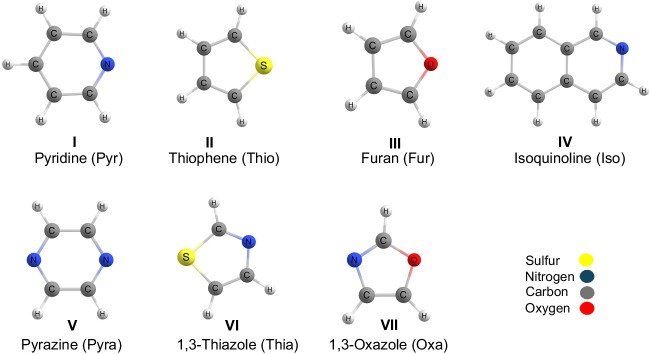
Chemical structures of heterocyclic aromatic model compounds and the respective abbreviations: (I) pyridine, Pyr; (II) thiophene, Thio; (III) furan, Fur; (IV) isoquinoline, Iso; (V) pyrazine, Pyra; (VI) 1,3-thiazole, Thia; and (VII) 1,3-oxazole, Oxa

In the present study, we explore the formation of bridges of water molecules spanning between the stacked homodimers of N-, O-, and S-containing heterocycles and evaluate the enhancement of the supramolecular interaction strength. The heterocycles pyridine, thiophene, isoquinoline, and furan were selected due to their presence as moieties in asphaltene structures [33–38]. Additionally, the heterocycles pyrazine, 1,3-oxazole, and 1,3-thiazole were included to study aromatic compounds with two heteroatoms. The structures of these compounds are shown in Fig. 2. The contributions of π-π stacking and hydrogen bonding interaction to the aggregate stabilization were investigated using non-covalent interaction and energy decomposition analysis (EDA). The interaction energy was rationalized in terms of geometric and energetic parameters inherent to the dimers and monomers.

## Computational methods

The DFT calculations were performed with the ωB97X-D exchange–correlation density functional with a dispersion energy correction [39]. Several studies have previously shown successful results with the account of the interaction of π-conjugated oligomers [40], aggregation of asphaltene model compounds [19, 32], and the interaction of polycondensed aromatic molecules [41]. We employed the Def2-SVP basis set for all atoms. This is a split-valence double-zeta basis set with polarization functions for all atoms, proposed by Ahlrichs and Weigeng [42]. All calculations were performed in Gaussian09 [43]. After full geometry optimization, the second-order force constant matrix was calculated to confirm that the optimized geometry is a genuine minimum on the potential energy surface. The thermodynamic results are important for the computational determination of the enthalpy and Gibbs free energy (at 1 atm and 298 K) of the formation of supramolecular aggregates. The basis set superposition error (BSSE) was accounted for by the counterpoise correction procedure [44]. The B3LYP/Def2-SVP method was employed for the energy decomposition analysis using the GAMESS software [45, 46]. The EDA procedure [47–49] decomposes the total interaction energy into five components: electrostatic (*E*_Elec_), polarization (*E*_Pol_), exchange (*E*_Xc_), dispersion (*E*_Disp_), and Pauli repulsion (*E*_Pauli_). The sum of the polarization and exchange terms yields the covalent component of the interaction.

Non-covalent interactions between the monomers in the dimer structure and between the monomers and water molecules in the aggregate structure were also analyzed. For this, we used the NCIplot software [50]. This software calculates the attractive and repulsive interactions present in the system as a function of the electron density and its reduced gradient. Non-covalent interactions are plotted as an isosurface map using Visual Molecular Dynamics [51].

## Results and discussion

### Geometry optimization

**Fig. 3:**
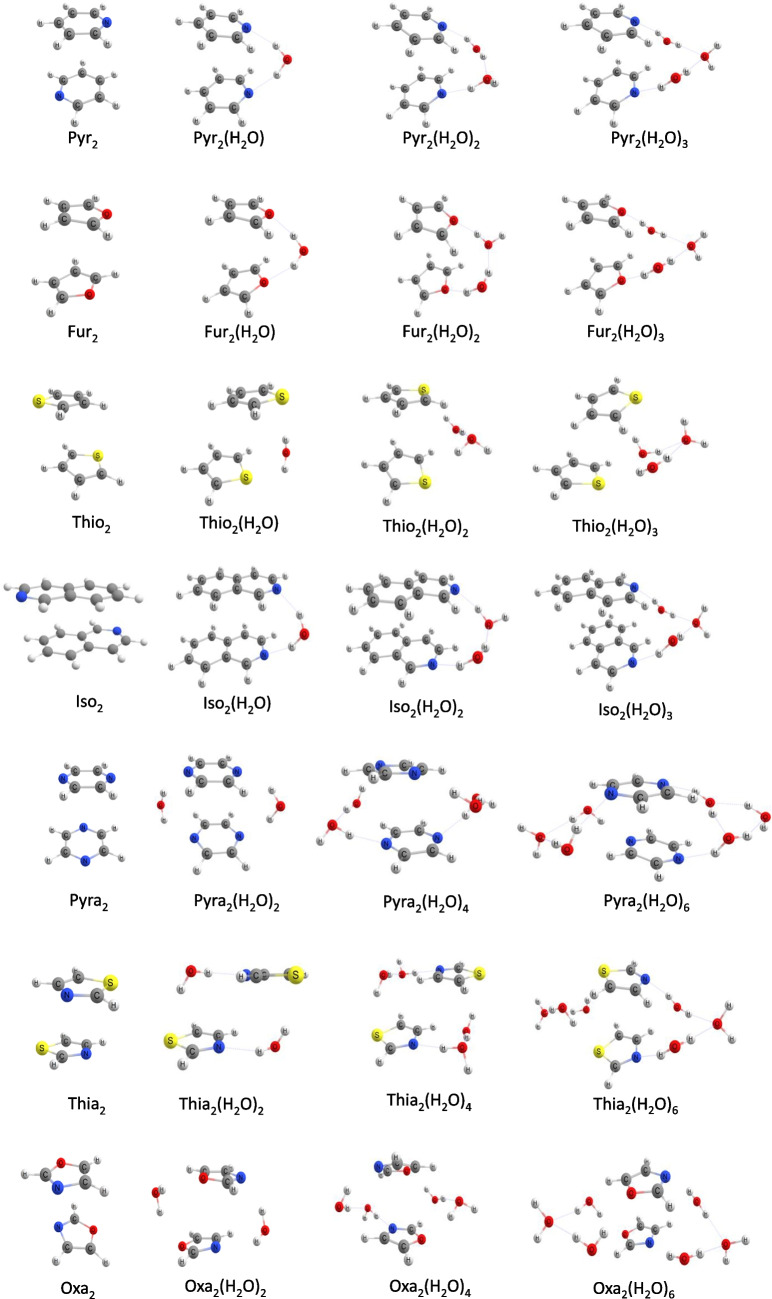
Optimized geometries of water-free dimers and aggregates containing one, two, or three water molecules per bridge

The geometries of 28 supramolecular aggregates containing homodimers and water molecules were fully optimized with the ωB97X-D/Def2-SVP method. The optimized structures for the aggregates containing dimers with and without water molecules are shown in Fig. 3. For pyridine, furan, thiophene, and isoquinoline which have one heteroatom, only one water bridge is formed, whereas for the pyrazine, oxazole, and thiazole rings that have two heteroatoms, two water bridges are formed, stabilizing the dimer structure.

For pyridine, furan, thiophene, isoquinoline, oxazole, and thiazole dimers, the bridge can be formed by one, two, or three water molecules, with the aromatic moieties retaining almost parallel orientation. For the pyrazine dimer, only bridges with one or two water molecules are formed. The pyrazine aggregate, composed of three water molecules (Pyra_2_(H_2_O)_6_), upon optimization converges to a structure where only two water molecules are in the bridge with the third water molecule being hydrogen bonded to the water bridge. As pyrazine has two heteroatoms, two water bridges may be formed, making the aggregate more rigid and keeping the interplanar distance short because of the stabilizing effects of the water bridges. The high symmetry of the pyrazine ring strengthens the π-stacking interaction and does not allow the aromatic rings to be sufficiently far from each other to accommodate a third water molecule in the bridge.

For isoquinoline, both the dimer without any water molecule (Iso_2_) and the aggregate with three water molecules (Iso_2_(H_2_O)_6_) have aromatic rings that are in non-parallel planes, whereas the aggregates of isoquinoline with one (Iso_2_(H_2_O)_2_) or two (Iso_2_(H_2_O)_4_) water molecules in the bridge have well-aligned aromatic planes. The bridge of one or two water molecules forces the aromatic planes to be one above the other. The dimer without water (Iso_2_) does not exhibit this effect. The aggregate with three water molecules has more degrees of freedom and a longer bridge that causes the distortion of the heterocyclic plane rather than constraining those to a parallel configuration.

The thiazole aggregates with one or two water molecules in the bridge exhibit more substantial horizontal displacement than the structures with three water molecules (Thia_2_(H_2_O)_6_) and the water-free dimer (Thia_2_). This is related to the interaction of the water molecules with the heteroatom of the aromatic system and the resultant competition between π-π stacking and electrostatic interaction. Moreover, in the aggregates with one (Thia_2_(H_2_O)_2_) or two (Thia_2_(H_2_O)_4_) water molecules per bridge as well as in the water-free dimer (Thia_2_), the heterocycles are in a staggered configuration; i.e., the N and S atoms of one of the monomers are on opposite sides from those in the other monomer. Thus, the water bridges span between different heteroatoms, forming N⋯H_2_O⋯S networks, whereas for the bridge containing three water molecules, the interaction is with the same heteroatom, forming N⋯H_2_O⋯N and S⋯H_2_O⋯S hydrogen bonding networks. We also calculated the thiazole aggregates with bridges containing one (Thia_2_(H_2_O)_2_) and two (Thia_2_(H_2_O)_4_) water molecules involving the same heteroatom, i.e., N⋯H_2_O⋯N and S⋯H_2_O⋯S, but these are less stable than the Thia_2_(H_2_O)_2_ and Thia_2_(H_2_O)_4_ shown in Fig. 3. As S has a larger atomic radius than N, its orbitals do not align with the one of the other atoms of the aromatic ring and the π-stacking interaction is more effective by the side of the N atom. This is the reason why the aromatic rings are horizontally displaced, as in the dimer without water molecules (Thia_2_) the S atoms are in positions opposite to each other. In the aggregate containing three water molecules per bridge (Thia_2_(H_2_O)_6_), the longer bridge allows the water trimer to interact with the same heteroatoms. It was not possible to optimize the aggregate with a water trimer bridge spanning between different heteroatoms.

### Energy calculation

The stabilization of the dimer structures is due to the π-π stacking and the water bridge hydrogen bond interactions. The π-π stacking interaction between the rings in each dimer structure was accounted for by means of the ASM method (activation strain model). This model, proposed by Fernández and Bickelhaupt [52], is a computational approach that uses electronic structure calculations to rationalize the factors that control stability in each stationary point along a reaction coordinate. According to the ASM model, the binding energy (*E*_BIND_) can be decomposed into two contributions along the reaction coordinate (Eq. 1). The first one is the strain or distortion energy, $\Delta {E}_{{\text{DEF}}}$, related to the deformation of the reactants, mainly affected by the rigidity of their molecular structure and by distortion in pendant groups. The second component is the interaction energy, $\Delta {E}_{{\text{INT}}}$, between the reactants, related to the binding capacity between the reactants, which, in our case, accounts to the π-π stacking interaction.

1 $$\Delta {E}_{BIND}=\Delta {E}_{DEF}+\Delta {E}_{INT}$$

In general, $\Delta {E}_{{\text{DEF}}}$ is a positive term, which destabilizes the system, and the $\Delta {E}_{{\text{INT}}}$ is a negative term, stabilizing the system. The $\Delta {E}_{{\text{INT}}}$ can be decomposed into stabilizing electrostatic interaction (coulomb) between fragments ∆*V*_*elst*_; destabilizing interaction derived from the overlap of filled orbitals ∆*E*_*Pa*u*li*_; orbital interaction energy ∆*E*_*oi*_, responsible for the charge transfer (HOMO–LUMO interactions, for example) and polarization (mixing unoccupied and occupied orbitals in the different fragments); and the interaction due to dispersion forces ∆*E*_*disp*_. These data are presented in the Supplementary Material. Table 1 presents the values of $\Delta {E}_{{\text{INT}}}$ between the heteroaromatic ring dimers and the interplanar distance of the structures shown in Fig. 3.

**Table 1:** $\Delta {E}_{{\text{INT}}}$ (in kcal mol^−1^), Δ*H* (in kcal mol^−1^), and Δ*G* (in kcal mol^−1^) for the formation of the aggregates presented in Fig. 3. The distance (*D*) between the centers of the aromatic rings is in angstrom (Å). Values for $\Delta {E}_{{\text{INT}}}$, Δ*H*, and Δ*G* are corrected by BSSE

	$\Delta {E}_{{\text{INT}}}$	ΔH*	ΔG*	D**
Pyr_2_	− 5.53	− 2.99	3.79	3.630
Pyr_2_(H_2_O)	− 2.93	− 7.80	10.92	3.707
Pyr_2_(H_2_O)_2_	− 5.03	− 13.15	9.60	3.727
Pyr_2_(H_2_O)_3_	− 2.94	− 10.16	10.41	3.749
Fur_2_	− 2.47	− 1.19	7.55	3.467
Fur_2_(H_2_O)	− 2.21	− 0.18	20.48	3.637
Fur_2_(H_2_O)_2_	− 2.37	− 3.49	20.18	3.700
Fur_2_(H_2_O)_3_	− 2.33	− 0.98	21.26	3.707
Thio_2_	− 2.72	− 1.23	7.99	3.712
Thio_2_(H_2_O)	− 2.25	− 1.30	16.94	3.886
Thio_2_(H_2_O)_2_	− 2.68	− 4.32	16.60	3.785
Thio_2_(H_2_O)_3_	− 2.47	− 1.92	18.39	4.250
Iso_2_	− 8.60	− 7.10	3.71	3.624
Iso_2_(H_2_O)	− 6.88	− 11.76	9.20	3.626
Iso_2_(H_2_O)_2_	− 8.08	− 18.06	7.51	3.537
Iso_2_(H_2_O)_3_	− 8.25	− 15.34	8.38	3.547
Pyra_2_	− 4.25	− 3.63	7.07	3.508
Pyra_2_(H_2_O)_2_	− 2.93	− 12.92	15.36	3.600
Pyra_2_(H_2_O)_4_	− 3.99	− 24.23	13.94	3.448
Pyra_2_(H_2_O)_6_	− 3.75	− 27.28	8.26	3.582
Thia_2_	− 3.05	− 1.81	6.44	3.685
Thia_2_(H_2_O)_2_	− 1.17	− 6.85	21.30	4.810
Thia_2_(H_2_O)_4_	− 2.05	− 19.50	16.26	4.880
Thia_2_(H_2_O)_6_	− 3.20	− 11.14	21.43	3.723
Oxa_2_	− 3.21	− 1.86	7.65	3.437
Oxa_2_(H_2_O)_2_	− 2.29	− 8.40	18.24	3.511
Oxa_2_(H_2_O)_4_	− 3.09	− 19.59	17.59	3.393
Oxa_2_(H_2_O)_6_	− 3.04	− 12.90	19.75	3.380

*$\Delta {E}_{{\text{INT}}}$, Δ*H*, and Δ*G* values were calculated taking the isolated monomers and one, a cluster of two, or a cluster of three water molecules as reference

**The distance shown is the distance between the centers of the aromatic rings

The analysis of Table 1 shows that the strongest $\Delta {E}_{{\text{INT}}}$ interaction, corresponding to the lowest energy value, is for the aggregates without any water molecule in the structure. The larger freedom or reduced constraints of the π-electronic clouds of the rings that can undergo horizontal displacement enhance π-π stacking interaction. The largest value of $\Delta {E}_{{\text{INT}}}$ is for the isoquinoline ring, almost twice the values found for the other rings; it is followed by the pyrazole and pyridine rings. The isoquinoline is the only system with two fused rings, which enhances π-π stacking interaction. Thus, for water-free dimers, $\Delta {E}_{{\text{INT}}}$ is modulated by the size of the aromatic system.

The structures linked by water molecules are more rigid and constrained, which cannot assume the alignment between the ring planes required to accommodate the electron density for the π-stacking interaction. In all cases, we note that the aggregates containing a bridge of just one water molecule have the smallest (less negative) $\Delta {E}_{{\text{INT}}}$ interaction among the water-bridged dimers. The bridge formed by only one water molecule makes the system more rigid, restricting the planes from the adequate horizontal displacement for optimal π-stacking interaction. Generally, the aggregates with two water molecules in the bridge have the second lowest $\Delta {E}_{{\text{INT}}}$, followed by the dimers with three water molecules per bridge. The bridges containing two water molecules apparently provide more favorable structural arrangements for the aromatic planes that enhance the π-stacking interaction than the bridges with three water molecules.

In Table 1, we also show the interplanar distance in the dimers. For the pyridine and furan dimers, we notice that as the number of water molecules in the bridge increases, the distance becomes larger. This is due to the hydrogen bond interaction between the dimers and the water bridge that is so effective that enhances the distance between the aromatic planes. For the thiophene and pyrazole aggregates with bridges of two water molecules (Thio_2_(H_2_O)_2_ and Pyra_2_(H_2_O)_4_), the interplanar distance is smaller than that for the aggregates with bridges of three water molecules. This is attributable to the more effective accommodation of the aromatic electronic cloud overlap between the planes. For the oxazole and isoquinoline aggregates, the dimers with two (Oxa_2_(H_2_O)_2_ and Iso_2_(H_2_O)_2_) and three (Oxa_2_(H_2_O)_3_ and Iso_2_(H_2_O)_3_) water molecules in the bridge have shorter interplanar distances than those with one or without any water molecule in the bridge. For the isoquinoline system, the aromatic planes deviate from parallel to a crossed configuration as the number of water molecules increases, disrupting the π-π stacking interaction; however, the shorter interplanar distance likely partially offsets for the decreased parallel alignment. The thiazole dimer with two water molecules (Thio_2_(H_2_O)_4_) in the bridge has the longest interplanar distance of all aggregates analyzed. The π-π stacking interaction and dipole–dipole interaction of the S atom with the water molecules in the bridge are not as strong as the hydrogen bonding within the bridge.

The enthalpy (Δ*H*) and Gibbs free energy (Δ*G*) for the supramolecular aggregation were calculated considering either none or a cluster of 1–3 water molecules (Eqs. 2 and 3).

2 $$\Delta H={H}_{{\text{complex}}}-({2H}_{{\text{monomer}}}+{H}_{({{\text{H}}}_{2}{\text{O}})x})$$

3 $$\Delta G={G}_{{\text{complex}}}-({2G}_{{\text{monomer}}}+{G}_{\left({{\text{H}}}_{2}{\text{O}}\right)x})$$

where ${H ({\text{or}} G)}_{{\text{complex}}}$ is the enthalpy (or Gibbs free energy) of the aggregate with or without water molecules in the bridge, ${H ({\text{or}} G)}_{{\text{monomer}}}$ is the enthalpy (or Gibbs free energy) of each monomer, and ${E}_{{{({\text{H}}}_{2}{\text{O}})}_{x}}$ is the enthalpy (or Gibbs free energy) of one ($x=1$), cluster of two ($x=2$), or cluster of three ($x=3$) water molecules.

In Table 1, we show the π-π stacking interaction and thermodynamic results for all the structures shown in Fig. 3. In the water-free dimers, the Δ*H* and Δ*G* are stabilized only due to the π-π stacking interaction between the monomers. In aggregates with the water molecules, in addition to the π-π stacking interaction, the hydrogen bonds also help stabilize the complexes.

The analysis of Table 1 shows that the aggregations are exothermic (negative Δ*H*), although not spontaneous (positive Δ*G*). In general, the aggregates containing two or three water molecules per bridge are the ones that have the smallest values of Δ*H*, suggesting that the complexes formed with two water molecules are the most stable, followed by those with three water molecules. This result has previously been reported based on the calculation of the aggregation of model asphaltene compounds [32]. As the Δ*H* analysis accounts for both the π-stacking and the hydrogen bond terms, the aggregates with two water molecules per bridge must have the strongest stabilization by hydrogen bonding. The Δ*G* analysis shows that the structures without any water molecules have the smallest values, although still being positive, in agreement with the experimental report that these molecules do not aggregate spontaneously [31].

The water bridges increase the distance between the heterocycles, by a maximum of 0.1 Å, weakening the π-π stacking interaction; however, the stabilization of the aggregate seems to be compensated by the hydrogen bonds, since the formation of complexes with two or three water molecules is exothermic.

### Intermolecular interactions

**Fig. 4:**
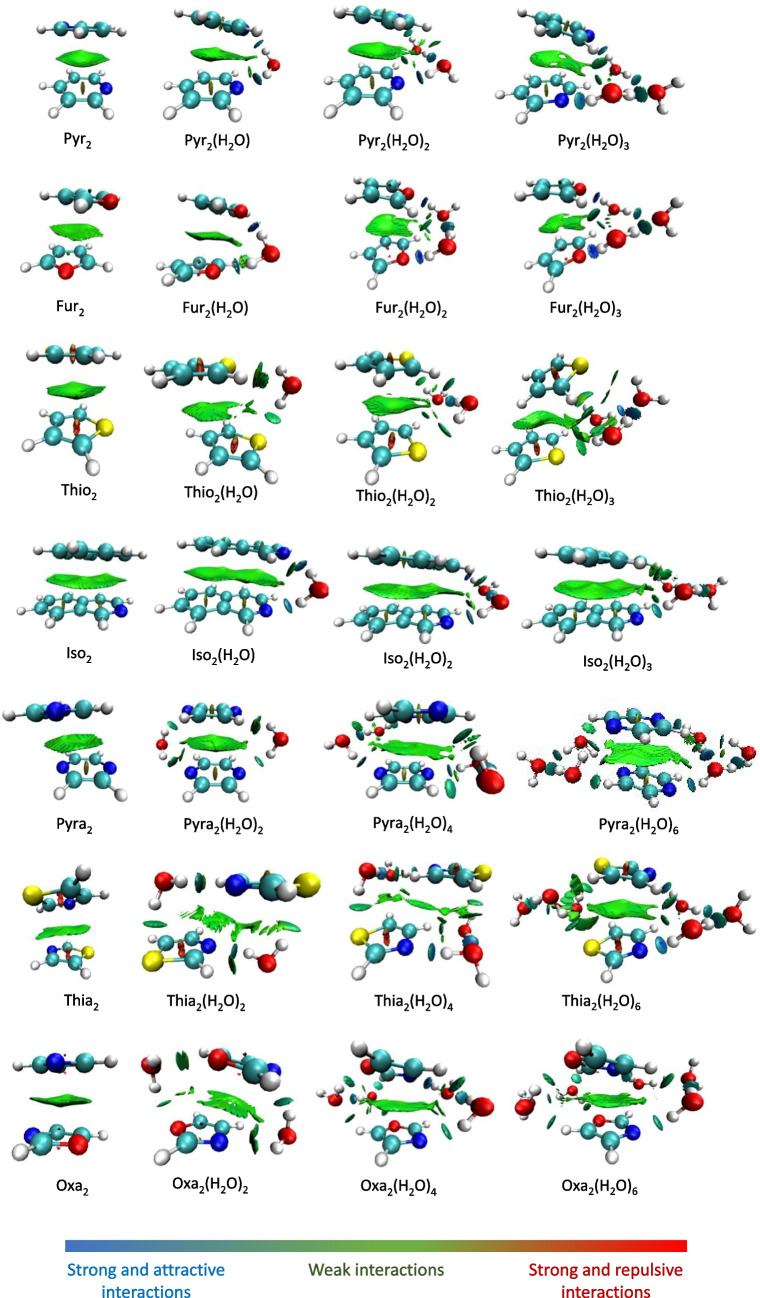
Optimized structures of the dimers without water, and with one, two, or three water molecules per bridge, showing the non-covalent interaction obtained with NCIplot

To examine the non-covalent interactions between the monomers in the dimer structure and between the monomers and water bridges, the NCI plots of the optimized structures of the supramolecular aggregates are presented in Fig. 4. The isosurface colors vary between green and blue colors. The green color represents a weak favorable non-covalent interaction, such as the van der Waals interaction. The blue color represents strong favorable non-covalent interactions, such as conventional hydrogen bonds. Unfavorable and repulsive interactions, represented in red color, are not observed on the isosurfaces of any of the aggregates investigated.

The analysis of Fig. 4 shows that the green area corresponds to the π-π stacking interaction between the heteroaromatic rings. The green color indicates that this interaction is weak, as expected for this type of interaction, and the dispersed isosurface shows a delocalized interaction. There is no significant difference in the plotted area for the aggregates with one, two, or three waters in relation to the dimers without water. As isoquinoline is composed by two fused aromatic rings, its π-stacking interaction is more diffuse and is seen with an enlarged green area. Light blue areas, representing strong attractive interactions due to hydrogen bonding, are also seen in Fig. 4. The blue regions represent localized interactions and are not as dispersed as the green ones. Furthermore, as the number of water molecules per bridge increases, the localized interactions shown in blue, corresponding to hydrogen bonds, increase, indicating a stronger bridging interaction, as expected for hydrogen bonding networks. For the interaction between the S atom and the water molecules, in thiophene and thiazole, we could not observe blue areas, indicating weaker dipole–dipole interactions.

### Energy decomposition analysis

The EDA method decomposes the total interaction energy (*E*_tot_) of supramolecular aggregates into five components: electrostatic, *E*_Elec_, (classical electrostatic interaction); polarization, *E*_Pol_, (orbital overlap); exchange, *E*_Xc_, (parallel spin stabilization); dispersion, *E*_Disp_, (long-range interactions); and Pauli repulsion, *E*_Pauli_, (electronic repulsion) terms [47–49]. The bond between the dimer (first fragment) and the water bridge (second fragment) was decomposed and analyzed. Table 2 shows the EDA results.

**Table 2:** The EDA components *E*_tot_, *E*_Elec_, *E*_Pol_, *E*_Xc_, *E*_Disp_, and *E*_Pauli_ in kcal mol^−1^

	*E*_Elec_	*E*_Pol_	*E*_Xc_	*E*_Disp_	*E*_Pauli_	*E*_tot_
Pyr_2_(H_2_O)	− 16.55	− 15.87	− 7.94	− 6.36	32.99	− 13.72
Pyr_2_(H_2_O)_2_	− 23.16	− 22.63	− 10.93	− 8.79	44.39	− 21.11
Pyr_2_(H_2_O)_3_	− 22.61	− 25.41	− 11.62	− 8.96	46.19	− 22.40
Fur_2_(H_2_O)	− 13.65	− 13.98	− 9.00	− 6.94	37.85	− 5.72
Fur_2_(H_2_O)_2_	− 23.61	− 22.57	− 15.49	− 10.28	60.47	− 11.48
Fur_2_(H_2_O)_3_	− 25.92	− 25.41	− 17.88	− 10.81	67.31	− 12.71
Thio_2_(H_2_O)	− 5.53	− 9.15	− 2.88	− 4.72	16.97	− 5.31
Thio_2_(H_2_O)_2_	− 9.38	− 14.22	− 4.82	− 6.81	25.52	− 9.71
Thio_2_(H_2_O)_3_	− 11.74	− 14.94	− 5.87	− 8.52	32.19	− 8.90
Iso_2_(H_2_O)	− 17.94	− 16.47	− 8.94	− 6.57	36.02	− 13.90
Iso_2_(H_2_O)_2_	− 30.95	− 29.23	− 18.33	− 10.47	67.80	− 21.18
Iso_2_(H_2_O)_3_	− 21.00	− 25.24	− 9.36	− 9.25	41.12	− 23.73
Pyra_2_(H_2_O)_2_	− 22.75	− 23.44	− 7.76	− 10.26	40.00	− 24.21
Pyra_2_(H_2_O)_4_	− 41.07	− 40.15	− 18.66	− 16.94	79.71	− 37.11
Pyra_2_(H_2_O)_6_	− 38.89	− 38.92	− 17.02	− 16.83	75.78	− 35.88
Thia_2_(H_2_O)_2_	− 19.50	− 21.94	− 9.29	− 10.21	44.65	− 16.29
Thia_2_(H_2_O)_4_	− 31.72	− 35.84	− 15.64	− 15.93	70.42	− 28.70
Thia_2_(H_2_O)_6_	− 34.94	− 37.34	− 18.73	− 16.91	80.49	− 27.43
Oxa_2_(H_2_O)_2_	− 16.39	− 18.86	− 4.37	− 8.93	29.54	− 19.01
Oxa_2_(H_2_O)_4_	− 35.35	− 34.25	− 14.10	− 15.47	66.22	− 32.95
Oxa_2_(H_2_O)_6_	− 35.91	− 37.39	− 14.56	− 15.88	67.48	− 36.26

The analysis of Table 2 shows a trend that is observed for all the components of the interaction as well as for the total interaction energy. As a general trend, the total interaction energy increases when increasing the number of water molecules in the bridge. However, the incremental difference is much more relevant for the first water molecule than for the second or the third one. For example, the difference in the *E*_tot_ values for the aggregates with one water molecule per bridge to those with two water molecules per bridge is 9.15 ± 3.55 kcal mol^−1^, whereas the difference for the aggregates with two water molecules per bridge to those with three water molecules per bridge is only 1.05 ± 1.65 kcal mol^−1^. This shows that the aggregates with two and three water molecules are significantly more stabilized than those with only one water molecule per bridge. Also, the energies of aggregates with two or three water molecules in the bridge do not vary substantially.

The dispersion term (Table 2), accounting for long-range interactions, has the smallest variation (standard deviation of 3.68 kcal mol^−1^). It also changes more strongly from the aggregates with one water molecule per bridge to the ones with two water molecules per bridge than for additional water molecules. The structures with the stronger electrostatic term also have the largest repulsion term (*E*_Pauli_).

**Fig. 5:**
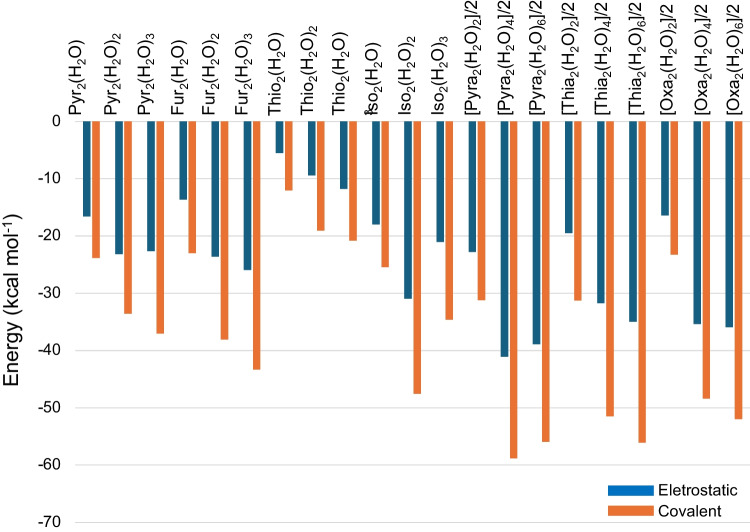
Covalent component (*E*_Pol_ and *E*_Xc_) in orange and electrostatic component (*E*_Elec_) in blue of the total interaction energy based on EDA

In Fig. 5, we present the electrostatic and covalent components of the total interaction energy. In this model, the ionic character of the interaction is accounted for by the *E*_Elec_ term, which comes mainly from opposite charge attraction sites. The covalent component is due to the sum of the *E*_Pol_ and *E*_Xc_ terms and considers the overlap of the atomic orbitals that compose the interaction. We can see for all dimers that the covalent character is almost two times larger than the ionic character, corresponding to a stabilization of − 12.54 ± 4.41 kcal mol^−1^. As the pyrazole, thiazole, and pyrazine aggregates have two water bridges, the stabilization per water bridge is the total value divided by 2. Considering the stabilization energy per water bridge, the most stable aggregates are those with hydrogen bonds between the heteroatom of the dimer and water of the bridge, i.e., interaction of H of water bridge with the O or N atom of the heterocycle. The S-containing heterocycles thiophene and thiazole with dipole–dipole interaction between the H atom of the water bridge and S atom of the heterocycle have lower stabilization energy.

## Conclusion

We investigated the interaction of the supramolecular aggregation of seven heterocyclic aromatic compounds as water-free dimers as well as dimers with water bridges bonded to the heteroatoms and spanning between the organic planes. We observed, for most of the aggregates, that the interaction is favored by bridges composed of two water molecules. Only for the 1,3-thiazole the favorite bridge has three water molecules, probably due to the softness of the sulfur atom. The π-π stacking interaction analysis showed that the water-free dimers have the strongest interaction, followed by the dimers with two water molecules per bridge. In almost all cases, the π-π stacking interaction strength is modulated by the interplanar distance between the monomers in the dimer structure. The Δ*H* analysis showed that aggregation is an exothermic process. The most stable aggregate for each heterocycle is the system with two water molecules per bridge. The Δ*G* analysis showed nonspontaneous aggregation processes with the smallest values for the dimer without any water molecule in the bridge. The NCI plot analysis identified strong interaction sites around the water molecules, representing hydrogen bonding interactions, and weak attraction between the planes of the organic molecules, representing the π-stacking interactions. The hydrogen bonds between the dimers and the water bridges were decomposed by using the EDA method. The results indicated that the covalent character (polarization and exchange) of the interaction is almost twice as large as the electrostatic term. We also noticed that one water molecule in the bridge led to a small stabilization of the aggregate, whereas two or three water molecules in the bridge add a considerable stabilization to the supramolecular system, with the aggregates having two water molecules per bridge being the most stable. Our findings justify the conclusion that the π-π stacking interaction is as important as hydrogen bonding for the stabilization of the dimers bridged by water molecules.

### Supplementary Information

Below is the link to the electronic supplementary material.

Supplementary file1 (DOCX 119 KB)

## Data Availability

The authors declare that the data supporting the findings of this study are available within the paper and its Supplementary Information files.
