# Supplementary material for: Density functional theory investigation of the contributions of π-π stacking and hydrogen bonding with water to the supramolecular aggregation interactions of model asphaltene heterocyclic compounds
Source: J Mol Model. 2024 Apr 24;30(5):145. doi: 10.1007/s00894-024-05922-3 (PMC11043155; doi:10.1007/s00894-024-05922-3)
Supplement: Supplementary file 1 — Supplementary file1 (DOCX 119 KB) [file 894_2024_5922_MOESM1_ESM.docx]

**Supporting Information**

for

**Density Functional Theory Investigation of the Contributions of π−π Stacking and Hydrogen Bonding with Water to the Supramolecular Aggregation Interactions of Asphaltene Heterocycles**

Milena D. Lessa^a^, Stanislav R. Stoyanov^b,^*, José Walkimar de M. Carneiro^a^, Leonardo M. da Costa^c,^*

**Supporting Information**

for

**Density Functional Theory Investigation of the Contributions of π−π Stacking and Hydrogen Bonding with Water to the Supramolecular Aggregation Interactions of Asphaltene Heterocycles**

Milena D. Lessa^a^, Stanislav R. Stoyanov^b,^*, José Walkimar de M. Carneiro^a^, Leonardo M. da Costa^c,^*

**SUMARY**

[1.$\boldsymbol{\Delta}\boldsymbol{E}_{\boldsymbol{INT}}$ **(in kcal mol^-1^) calculation to the formation of the aggregates presented in Figure 4 using the ASM method (Activation Strain Model)**. S3](#_Toc72332668)

[2. **Cartesian coordinates of the cluster of two water molecules end three water molecules** S5](#_Toc72332669)

[3. **Cartesian coordinates of the most stable aggregates containing water-free dimers and dimers containing one, two, or three water molecules per bridge (Figure 4)** S5](#_Toc72332670)

**1.** $\boldsymbol{\Delta}\boldsymbol{E}_{\boldsymbol{INT}}$ **(in kcal mol^-1^) calculation to the formation of the aggregates presented in Figure 4 using the ASM method (Activation Strain Model)**

The π-π stacking interaction between the rings in each dimer structure was accounted using the ASM method (Activation Strain Model). According to the ASM, the energy can be decomposed as shown in Equation S1. The $\Delta E_{BIND}$ and $\Delta E_{STRAIN}$ can be calculated using computational approaches, the $\Delta E_{INT}$ can be inferred by the Equation S2.

$\Delta E_{BIND}=\Delta E_{STRAIN}+\Delta E_{INT}$ Equation S1

$\Delta E_{INT}= \Delta E_{BIND}-(\Delta E_{STRAIN\left( frag1 \right)}+\Delta E_{STRAIN\left( frag2 \right)})$ Equation S2

We run single-points of the dimer as shown in a orange circle in Figure S1 (excluding the water cluster) corrected by BSSE, the Equation S3 was used to calculate $\Delta E_{BIND}$.

$\Delta E_{BIND}=E_{Dimer(single-point)}-{2E}_{monomer}$ Equation S3

We also run single-points of Fragment 1 and Fragment 2, separately. The $\Delta E_{STRAIN}$ was deduced by the Equation S4.

$\Delta E_{STRAIN(Fragment x)}=E_{Fragment x}-E_{monomer}$ Equation S4


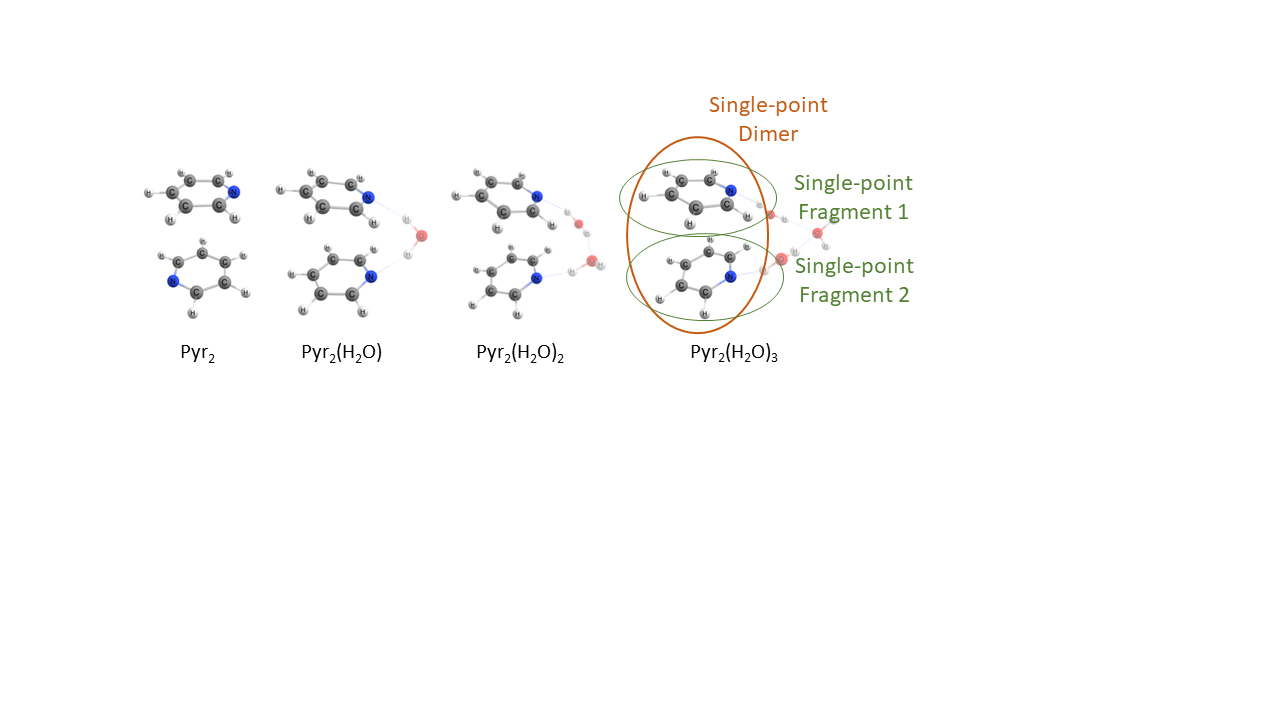


Figure S1. In orange the dimer used to calculate $\Delta E_{BIND}$ and in green the fragments to calculate the $\Delta E_{STRAIN}$.

Table S1. $\Delta E_{INT}$ (in kcal mol^-1^), $\Delta E_{STRAIN}$ (in kcal mol^-1^) and Δ$E_{BIND}$ (in kcal mol^-1^) to the formation of the aggregates presented in Figure 4. The Δ$E_{BIND}$ was corrected by BSSE.

|  | **Strain Energy** $\boldsymbol{\Delta}\boldsymbol{E}_{\boldsymbol{STRAIN}}$ | | **Interaction energy** $\boldsymbol{\Delta}\boldsymbol{E}_{\boldsymbol{INT}}$ | **Δ**$\boldsymbol{E}_{\boldsymbol{BIND}}$ |
| --- | --- | --- | --- | --- |
|  | **Fragment 1** | **Fragment 2** |  |  |
| **Pyr_2_** | 0.01 | 0.02 | -5.53 | -5.50 |
| **Pyr_2_(H_2_O)** | 0.11 | 0.05 | -2.93 | -2.77 |
| **Pyr_2_(H_2_O)_2_** | 0.27 | 0.13 | -5.03 | -4.63 |
| **Pyr_2_(H_2_O)_3_** | 0.33 | 0.10 | -2.94 | -2.51 |
| **Fur_2_** | 0.01 | 0.01 | -2.47 | -2.45 |
| **Fur_2_(H_2_O)** | 0.09 | 0.08 | -2.21 | -2.04 |
| **Fur_2_(H_2_O)_2_** | 0.36 | 0.15 | -2.37 | -2.88 |
| **Fur_2_(H_2_O)_3_** | 0.29 | 0.29 | -2.33 | -1.75 |
| **Thio_2_** | 0.01 | 0.01 | -2.72 | -2.70 |
| **Thio_2_(H_2_O)** | 0.04 | 0.11 | -2.25 | -2.09 |
| **Thio_2_(H_2_O)_2_** | 0.26 | 0.06 | -2.68 | -2.36 |
| **Thio_2_(H_2_O)_3_** | 0.33 | 0.08 | -2.47 | -2.06 |
| **Iso_2_** | 0.02 | 0.02 | -8.60 | -8.56 |
| **Iso_2_(H_2_O)** | 0.17 | 0.06 | -6.88 | -6.65 |
| **Iso_2_(H_2_O)_2_** | 0.50 | 0.13 | -8.08 | -7.45 |
| **Iso_2_(H_2_O)_3_** | 0.29 | 0.37 | -8.25 | -7.59 |
| **Pyra_2_** | 0.01 | 0.01 | -4.25 | -4.23 |
| **Pyra_2_(H_2_O)_2_** | 0.17 | 0.17 | -2.93 | -2.58 |
| **Pyra_2_(H_2_O)_4_** | 0.25 | 0.64 | -3.99 | -3.10 |
| **Pyra_2_(H_2_O)_6_** | 0.52 | 0.29 | -3.75 | -2.94 |
| **Thia_2_** | 0.01 | 0.01 | -3.05 | -3.03 |
| **Thia_2_(H_2_O)_2_** | 0.13 | 0.12 | -1.17 | -0.91 |
| **Thia_2_(H_2_O)_4_** | 0.34 | 0.35 | -2.05 | -1.36 |
| **Thia_2_(H_2_O)_6_** | 0.27 | 0.28 | -3.20 | -2.65 |
| **Oxa_2_** | 0.02 | 0.03 | -3.21 | -3.16 |
| **Oxa_2_(H_2_O)_2_** | 0.13 | 0.15 | -2.29 | -2.01 |
| **Oxa_2_(H_2_O)_4_** | 0.27 | 0.81 | -3.09 | -2.01 |
| **Oxa_2_(H_2_O)_6_** | 0.83 | 0.71 | -3.04 | -1.50 |

**2. Cartesian coordinates of the cluster of two water molecules end three water molecules**

**Cluster of two water molecules**

8 1.383185000 -0.113469000 -0.055924000

1 1.588795000 0.729678000 -0.473853000

1 1.542290000 0.045372000 0.880700000

8 -1.473470000 0.109754000 0.049900000

1 -1.883989000 -0.683421000 -0.304127000

1 -0.524813000 -0.061909000 -0.054530000

E_SCF_ = -152.6870274 a.u.

**Cluster of three water molecules**

8 -0.930863000 1.415519000 -0.037833000

1 -1.250413000 0.776091000 0.609974000

1 0.011420000 1.201397000 -0.072335000

8 -0.931251000 -1.415165000 0.037985000

1 -1.250041000 -0.776663000 -0.611097000

1 0.011398000 -1.202536000 0.071802000

8 1.620568000 -0.000078000 -0.000090000

1 2.205855000 -0.055094000 -0.762522000

1 2.204150000 0.054598000 0.763676000

E_SCF_ = -229.040262 a.u.

**3. Cartesian coordinates of the most stable aggregates containing water-free dimers and dimers containing one, two, or three water molecules per bridge (Figure 4)**

**(Pyr)_2_**

6 -1.706684000 1.352911000 -0.500772000

6 -1.115896000 0.464542000 -1.394997000

6 -1.226745000 -0.902071000 -1.141001000

7 -1.865018000 -1.406253000 -0.086275000

6 -2.426992000 -0.548578000 0.763206000

6 -2.380267000 0.836519000 0.603223000

1 -1.635875000 2.431187000 -0.660096000

1 -0.564733000 0.821654000 -2.266188000

1 -0.768688000 -1.624838000 -1.824996000

1 -2.945769000 -0.985178000 1.624153000

1 -2.860505000 1.492143000 1.332093000

6 1.381867000 -1.101805000 1.010254000

6 1.114397000 0.180237000 1.480289000

6 1.539860000 1.268618000 0.718246000

7 2.194277000 1.149728000 -0.435001000

6 2.453306000 -0.081468000 -0.872971000

6 2.070242000 -1.237112000 -0.193133000

1 1.047184000 -1.981396000 1.563768000

1 0.574521000 0.339700000 2.415138000

1 1.335655000 2.289770000 1.059365000

1 2.993925000 -0.156972000 -1.823179000

1 2.300953000 -2.221153000 -0.605185000

E_SCF_ = -496.0417465 a.u.

**Pyr_2_(H_2_O)**

6 -1.656243000 -1.896275000 0.415398000

6 -1.157919000 -1.042032000 1.395624000

6 -1.307895000 0.331339000 1.215326000

7 -1.909203000 0.865371000 0.153177000

6 -2.391254000 0.044465000 -0.778138000

6 -2.289774000 -1.343375000 -0.695207000

1 -1.550384000 -2.978829000 0.515335000

1 -0.652578000 -1.428779000 2.281850000

1 -0.908180000 1.040605000 1.947045000

1 -2.875301000 0.516644000 -1.639267000

1 -2.696730000 -1.973444000 -1.487971000

8 -0.298928000 3.232068000 -0.146849000

1 0.465609000 2.712806000 0.152662000

1 -1.029670000 2.595702000 -0.082166000

6 2.111615000 -1.516813000 -0.630680000

6 2.601150000 -1.010452000 0.571187000

6 2.311294000 0.313215000 0.899294000

7 1.585445000 1.112388000 0.120340000

6 1.123624000 0.626130000 -1.032420000

6 1.361587000 -0.680330000 -1.453516000

1 2.313463000 -2.550210000 -0.921872000

1 3.198479000 -1.627243000 1.244944000

1 2.678314000 0.746392000 1.835716000

1 0.520790000 1.322498000 -1.623558000

1 0.956808000 -1.032231000 -2.403750000

E_SCF_ = -572.4007298 a.u.

**Pyr_2_(H_2_O)_2_**

6 0.257136000 2.209949000 -1.008553000

6 -0.364276000 1.120030000 -1.608558000

6 -1.501327000 0.582566000 -1.005484000

7 -2.032189000 1.077848000 0.113236000

6 -1.444323000 2.134357000 0.674477000

6 -0.298064000 2.734644000 0.157149000

1 1.160614000 2.645625000 -1.441111000

1 0.034307000 0.669230000 -2.518677000

1 -1.975000000 -0.312954000 -1.420417000

1 -1.903592000 2.516887000 1.592054000

1 0.150348000 3.591406000 0.663265000

8 -1.535889000 -2.630358000 -1.027291000

1 -0.612905000 -2.378687000 -0.791381000

1 -1.554258000 -3.589433000 -0.966056000

6 2.703629000 0.258523000 0.893314000

6 2.943431000 -0.351267000 -0.336201000

6 2.015736000 -1.275925000 -0.808221000

7 0.902652000 -1.587801000 -0.145579000

6 0.664703000 -0.996300000 1.029321000

6 1.544941000 -0.071454000 1.589647000

1 3.409672000 0.987270000 1.298389000

1 3.837428000 -0.121985000 -0.918750000

1 2.170111000 -1.778635000 -1.768527000

1 -0.295171000 -1.239209000 1.504399000

1 1.309305000 0.388842000 2.550223000

8 -2.469658000 -1.530362000 1.432478000

1 -2.612802000 -0.612356000 1.152773000

1 -2.296438000 -1.981292000 0.587364000

E_SCF_ = -648.7652054 a.u.

**Pyr_2_(H_2_O)_3_**

6 2.293892000 -1.590406000 1.162381000

6 1.146491000 -0.962956000 1.636237000

6 -0.063716000 -1.193713000 0.981824000

7 -0.167617000 -2.002748000 -0.075153000

6 0.932214000 -2.608448000 -0.521964000

6 2.186853000 -2.434032000 0.057834000

1 3.260844000 -1.423307000 1.642725000

1 1.176038000 -0.283732000 2.489642000

1 -0.966428000 -0.658304000 1.301754000

1 0.809315000 -3.259492000 -1.393909000

1 3.058854000 -2.945921000 -0.352997000

8 -2.288130000 1.045971000 1.793884000

1 -1.654248000 1.533645000 1.235181000

1 -2.977165000 0.771785000 1.173081000

6 2.291497000 1.591640000 -1.163183000

6 2.184785000 2.435056000 -0.058434000

6 0.930382000 2.609134000 0.521958000

7 -0.169505000 2.003144000 0.075657000

6 -0.065893000 1.194271000 -0.981456000

6 1.144019000 0.964022000 -1.636610000

1 3.258276000 1.424822000 -1.643969000

1 3.056878000 2.947022000 0.352105000

1 0.807737000 3.260050000 1.394035000

1 -0.968495000 0.658381000 -1.300877000

1 1.173273000 0.284982000 -2.490172000

8 -2.287332000 -1.047923000 -1.793188000

1 -1.652693000 -1.534704000 -1.234528000

1 -2.976970000 -0.775395000 -1.172326000

8 -4.315947000 -0.001524000 0.000149000

1 -4.899812000 -0.598170000 0.479940000

1 -4.897423000 0.595967000 -0.481498000

E_SCF_ = -725.1139881 a.u.

**(Fur)_2_**

6 -1.347742000 0.508319000 -1.195611000

6 -1.939225000 1.175813000 -0.074357000

6 -2.249500000 0.196735000 0.818433000

8 -1.900063000 -1.011520000 0.334468000

6 -1.352285000 -0.815493000 -0.882302000

1 -0.959960000 0.956785000 -2.106383000

1 -2.106614000 2.242720000 0.050496000

1 -2.710742000 0.206154000 1.801982000

1 -1.000496000 -1.702618000 -1.400650000

6 1.938795000 1.175914000 0.076074000

6 1.347940000 0.506478000 1.196500000

6 1.352835000 -0.816860000 0.881228000

8 1.900224000 -1.010836000 -0.336011000

6 2.249016000 0.198267000 -0.818306000

1 2.105972000 2.243023000 -0.047350000

1 0.960325000 0.953411000 2.108097000

1 1.001326000 -1.704884000 1.398209000

1 2.709899000 0.209213000 -1.802013000

E_SCF_ = -459.5623594 a.u.

**Fur_2_(H_2_O)**

8 0.178616000 2.813464000 0.083500000

1 -0.576267000 2.310172000 -0.249239000

1 0.887918000 2.152872000 0.092829000

6 -1.657257000 -1.014107000 1.108652000

6 -2.317727000 -1.324566000 -0.126714000

6 -2.273558000 -0.188195000 -0.870967000

8 -1.638971000 0.791492000 -0.188104000

6 -1.267389000 0.283015000 1.010659000

1 -1.491457000 -1.674552000 1.955774000

1 -2.768844000 -2.269968000 -0.417862000

1 -2.637570000 0.072309000 -1.860593000

1 -0.730528000 0.970953000 1.657220000

6 2.080004000 -1.490362000 0.348775000

6 1.449455000 -1.351747000 -0.932709000

6 1.307081000 -0.017363000 -1.137736000

8 1.801049000 0.672533000 -0.082773000

6 2.265402000 -0.227971000 0.815073000

1 2.356157000 -2.412364000 0.854142000

1 1.139292000 -2.144088000 -1.608735000

1 0.884383000 0.581203000 -1.939231000

1 2.695294000 0.181331000 1.724515000

E_SCF_ = -535.9096724 a.u.

**Fur_2_(H_2_O)_2_**

8 -0.620041000 2.598262000 -1.088688000

1 -1.178653000 1.843669000 -0.815658000

1 -1.215949000 3.347877000 -1.168348000

8 0.869759000 2.374877000 1.342034000

1 1.432556000 1.662800000 0.991745000

1 0.411608000 2.683194000 0.543044000

6 -2.240174000 -1.742539000 0.177621000

6 -1.445623000 -1.269214000 1.275511000

6 -1.196835000 0.043032000 1.033043000

8 -1.788730000 0.409560000 -0.136588000

6 -2.412509000 -0.679766000 -0.649241000

1 -2.633073000 -2.745073000 0.026975000

1 -1.095692000 -1.838955000 2.132237000

1 -0.604920000 0.822532000 1.517899000

1 -2.931903000 -0.538647000 -1.592523000

6 1.291578000 -1.226580000 -1.352008000

6 1.934460000 -1.912781000 -0.268289000

6 2.324725000 -0.950120000 0.606896000

8 1.974441000 0.271415000 0.140982000

6 1.344292000 0.096095000 -1.047155000

1 0.841912000 -1.665224000 -2.238933000

1 2.083895000 -2.983745000 -0.157077000

1 2.840375000 -0.969315000 1.562463000

1 0.966920000 0.999206000 -1.522013000

E_SCF_ = -612.2701524 a.u.

**Fur_2_(H_2_O)_3_**

8 -1.851250000 1.012668000 1.667424000

1 -1.231813000 1.417539000 1.033924000

1 -2.594967000 0.720263000 1.122708000

8 -1.845283000 -1.027047000 -1.658922000

1 -1.222154000 -1.426104000 -1.024554000

1 -2.585815000 -0.728204000 -1.113395000

8 -3.996436000 0.002355000 -0.004368000

1 -4.585624000 -0.622083000 0.431767000

1 -4.572030000 0.620702000 -0.466574000

6 2.215256000 2.180133000 -0.354505000

6 1.690692000 1.314918000 -1.372534000

6 0.367075000 1.166819000 -1.105566000

8 0.042394000 1.883553000 0.002136000

6 1.167519000 2.488815000 0.452498000

1 3.239895000 2.525267000 -0.239666000

1 2.232747000 0.852169000 -2.193078000

1 -0.437291000 0.569330000 -1.540411000

1 1.059486000 3.097542000 1.345453000

6 2.221902000 -2.171264000 0.351599000

6 1.694335000 -1.311893000 1.373066000

6 0.369801000 -1.169053000 1.108052000

8 0.047335000 -1.883766000 -0.001764000

6 1.174887000 -2.482250000 -0.455381000

1 3.248093000 -2.510963000 0.234524000

1 2.235129000 -0.849344000 2.194552000

1 -0.437250000 -0.577293000 1.545713000

1 1.068701000 -3.088261000 -1.350393000

E_SCF_ = -688.6196392a.u.

**(Thio)_2_**

6 -1.236140000 1.231389000 -0.865810000

6 -1.988608000 1.457413000 0.326343000

6 -2.557399000 0.311607000 0.810390000

6 -1.255207000 -0.078590000 -1.254641000

1 -0.691690000 2.006788000 -1.404663000

1 -2.103626000 2.431235000 0.803178000

1 -3.182309000 0.194721000 1.694345000

1 -0.754572000 -0.531190000 -2.108498000

6 1.092028000 -0.600902000 1.325069000

6 1.540055000 -1.515905000 0.325523000

6 2.291912000 -0.908213000 -0.641723000

6 1.517109000 0.676578000 1.090710000

1 0.466760000 -0.878761000 2.173373000

1 1.307915000 -2.581153000 0.324516000

1 2.760387000 -1.363703000 -1.512724000

1 1.311299000 1.568968000 1.679105000

16 -2.183769000 -1.045945000 -0.178453000

16 2.462727000 0.777997000 -0.343035000

E_SCF_ = -1105.4311169 a.u.

**Thio_2_(H_2_O)**

8 0.641005000 2.732194000 0.615925000

1 -0.018920000 2.892940000 -0.067065000

1 1.187016000 2.033974000 0.227899000

6 -2.183577000 -0.672710000 1.240581000

6 -2.817587000 -1.151412000 0.052819000

6 -2.574316000 -0.352420000 -1.031090000

6 -1.470999000 0.474182000 1.029219000

1 -2.253235000 -1.164188000 2.211284000

1 -3.434610000 -2.049629000 0.008041000

1 -2.938291000 -0.475558000 -2.049791000

1 -0.866704000 1.062950000 1.716465000

6 1.412306000 -1.450874000 1.025639000

6 0.894789000 -1.734929000 -0.276152000

6 1.422723000 -0.922743000 -1.237154000

6 2.321553000 -0.433172000 1.019725000

1 1.109358000 -1.977964000 1.930282000

1 0.142318000 -2.496049000 -0.482051000

1 1.201726000 -0.911448000 -2.302982000

1 2.862092000 -0.006707000 1.863038000

16 -1.570464000 0.979535000 -0.610043000

16 2.561205000 0.189128000 -0.572710000

E_SCF_ = -1181.7781896 a.u.

**Thio_2_(H_2_O)_2_**

8 0.425936000 2.689113000 1.308617000

1 1.042547000 1.944393000 1.248567000

1 0.963511000 3.432857000 1.598643000

8 -0.455309000 2.704844000 -1.420275000

1 -1.152367000 2.039507000 -1.358774000

1 -0.191538000 2.823243000 -0.492172000

6 1.781570000 -2.057230000 -0.378674000

6 1.052132000 -1.193809000 -1.255439000

6 1.367330000 0.122562000 -1.085766000

6 2.629831000 -1.377212000 0.447647000

1 1.676083000 -3.142565000 -0.364553000

1 0.304447000 -1.538994000 -1.969004000

1 0.921954000 1.006183000 -1.550605000

1 3.311571000 -1.780334000 1.194645000

6 -1.201511000 -1.101151000 1.409547000

6 -1.780019000 -2.017257000 0.478168000

6 -2.510125000 -1.390273000 -0.491921000

6 -1.512742000 0.199079000 1.130510000

1 -0.567770000 -1.399580000 2.244694000

1 -1.654135000 -3.099147000 0.528215000

1 -3.060234000 -1.839697000 -1.316996000

1 -1.186313000 1.104164000 1.641771000

16 -2.511696000 0.317582000 -0.268040000

16 2.565972000 0.319296000 0.140815000

E_SCF_ = -1258.1362985 a.u.

**Thio_2_(H_2_O)_3_**

8 0.888461000 1.259075000 1.521976000

1 1.052897000 0.383865000 1.144398000

1 1.732787000 1.711202000 1.365797000

8 1.034799000 2.389146000 -1.287877000

1 0.376118000 2.176406000 -0.611191000

1 1.860356000 2.393820000 -0.778156000

8 3.228275000 2.144243000 0.409367000

1 3.720822000 1.331835000 0.238613000

1 3.888404000 2.833059000 0.540193000

6 0.382288000 -2.759548000 -0.052935000

6 -0.018094000 -1.677318000 -0.898216000

6 0.966359000 -0.748631000 -1.072237000

6 1.660524000 -2.627543000 0.410020000

1 -0.262352000 -3.599839000 0.206270000

1 -1.014390000 -1.573117000 -1.330042000

1 0.929973000 0.218474000 -1.580307000

1 2.218793000 -3.300510000 1.058333000

6 -3.559984000 -0.583818000 -0.218835000

6 -2.887971000 -0.733821000 1.033209000

6 -1.962731000 0.248981000 1.252517000

6 -3.126990000 0.512202000 -0.915909000

1 -4.334785000 -1.259497000 -0.582915000

1 -3.082120000 -1.541442000 1.739535000

1 -1.281851000 0.387640000 2.089895000

1 -3.469590000 0.868303000 -1.886263000

16 -1.905392000 1.362595000 -0.053272000

16 2.388657000 -1.184653000 -0.195951000

E_SCF_ = -1334.4864447 a.u.

**(Iso)_2_**

1 -3.223306000 -0.250164000 -1.580237000

6 -2.796160000 -0.049145000 -0.589551000

6 -2.800099000 -0.445555000 1.656984000

6 -1.747304000 0.404234000 1.889640000

6 -1.159776000 1.097882000 0.799513000

6 -1.706789000 0.862423000 -0.488995000

6 -1.153531000 1.519820000 -1.618512000

6 -0.096931000 2.383328000 -1.464278000

6 0.452018000 2.617070000 -0.178608000

6 -0.063716000 1.991576000 0.929975000

1 -3.268883000 -0.985968000 2.485679000

1 -1.360331000 0.547675000 2.901046000

1 0.367476000 2.165149000 1.918215000

1 1.298151000 3.299315000 -0.073072000

1 0.331102000 2.887924000 -2.332965000

1 -1.576962000 1.322662000 -2.606154000

1 1.343051000 -0.645439000 -2.887470000

6 0.984489000 -1.068117000 -1.939723000

6 -0.508164000 -2.390117000 -0.829282000

6 0.059092000 -2.138853000 0.395313000

6 1.180597000 -1.273446000 0.475567000

6 1.664402000 -0.719657000 -0.738408000

6 2.781293000 0.155513000 -0.716006000

6 3.390055000 0.470025000 0.473844000

6 2.903264000 -0.075301000 1.688706000

6 1.825055000 -0.926545000 1.693639000

1 -1.384224000 -3.040321000 -0.908484000

1 -0.355923000 -2.587056000 1.299911000

1 1.449326000 -1.346635000 2.629745000

1 3.395344000 0.185157000 2.628579000

1 4.248810000 1.144367000 0.491286000

1 3.144902000 0.576408000 -1.656897000

7 -3.326779000 -0.675252000 0.428957000

7 -0.052548000 -1.862445000 -1.992969000

E_SCF_ = -803.0139714 a.u.

**Iso_2_(H_2_O)**

8 3.400001000 2.297524000 -1.123390000

1 3.591503000 1.406142000 -0.782998000

1 2.664567000 2.573409000 -0.550120000

1 1.867048000 0.470092000 -1.832407000

6 1.882204000 -0.307352000 -1.059791000

6 3.014627000 -1.259230000 0.690024000

6 1.998337000 -2.138643000 0.967983000

6 0.818656000 -2.109671000 0.178730000

6 0.766444000 -1.168301000 -0.883470000

6 -0.393065000 -1.088169000 -1.698650000

6 -1.461357000 -1.915651000 -1.460808000

6 -1.410982000 -2.857431000 -0.401929000

6 -0.300786000 -2.956809000 0.400356000

1 3.933204000 -1.258966000 1.284090000

1 2.091054000 -2.852318000 1.789503000

1 -0.265196000 -3.685802000 1.213364000

1 -2.269705000 -3.508759000 -0.224435000

1 -2.357743000 -1.847281000 -2.079899000

1 -0.423004000 -0.351428000 -2.504589000

1 -0.067939000 3.142060000 -1.183432000

6 -0.093522000 2.481955000 -0.307490000

6 0.996766000 1.611219000 1.509138000

6 -0.069078000 0.826936000 1.868538000

6 -1.253453000 0.861186000 1.086503000

6 -1.269252000 1.727499000 -0.039294000

6 -2.426319000 1.797317000 -0.858409000

6 -3.525839000 1.029398000 -0.563942000

6 -3.511586000 0.163178000 0.558197000

6 -2.404212000 0.077345000 1.365943000

1 1.927588000 1.592733000 2.082396000

1 -0.006191000 0.171712000 2.739495000

1 -2.392297000 -0.596946000 2.225051000

1 -4.393630000 -0.442415000 0.777992000

1 -4.417469000 1.082932000 -1.192105000

1 -2.427096000 2.468583000 -1.720931000

7 2.954905000 -0.343299000 -0.307620000

7 0.987923000 2.430072000 0.427101000

E_SCF_ = -879.3718725 a.u.

**Iso_2_(H_2_O)_2_**

8 -4.270524000 0.769835000 -0.818214000

1 -3.744628000 -0.072495000 -0.860506000

1 -5.191867000 0.497855000 -0.816384000

8 -3.358293000 1.336312000 1.841178000

1 -2.506287000 1.744907000 1.591371000

1 -3.812188000 1.263235000 0.983705000

1 -0.388260000 1.203589000 -2.563465000

6 -0.145535000 1.401851000 -1.517907000

6 -1.130726000 1.767605000 -0.636108000

6 0.332456000 1.909971000 1.124870000

6 1.437660000 1.526300000 0.316266000

6 1.188549000 1.257457000 -1.055347000

6 2.270645000 0.848577000 -1.881050000

6 3.532803000 0.719834000 -1.354415000

6 3.778573000 0.990400000 0.015379000

6 2.750746000 1.384466000 0.835633000

1 -2.176669000 1.836742000 -0.951126000

1 0.496852000 2.112915000 2.190098000

1 2.927325000 1.588371000 1.894580000

1 4.788909000 0.880044000 0.414551000

1 4.359800000 0.406184000 -1.995493000

1 2.082989000 0.640277000 -2.936979000

1 0.110558000 -2.512126000 -2.436479000

6 -0.447937000 -2.116152000 -1.585786000

6 -1.762839000 -1.754053000 -1.725544000

6 -1.945335000 -1.086223000 0.466682000

6 -0.600186000 -1.450421000 0.741135000

6 0.187159000 -1.958049000 -0.324830000

6 1.554070000 -2.257755000 -0.084836000

6 2.092860000 -2.065051000 1.164275000

6 1.299995000 -1.572893000 2.231439000

6 -0.022355000 -1.267091000 2.024703000

1 -2.273865000 -1.857075000 -2.686918000

1 -2.536885000 -0.577125000 1.240301000

1 -0.645451000 -0.869948000 2.829483000

1 1.751253000 -1.429401000 3.215327000

1 3.147447000 -2.287711000 1.339915000

1 2.170039000 -2.633980000 -0.904510000

7 -0.892114000 2.020480000 0.678104000

7 -2.501050000 -1.228492000 -0.715038000

E_SCF_ = -955.7370666 a.u.

**Iso_2_(H_2_O)_3_**

8 2.715423000 -1.632282000 1.847213000

1 2.179005000 -1.880113000 1.072068000

1 3.587881000 -1.460529000 1.467008000

8 3.849604000 0.414443000 -1.884387000

1 3.307355000 1.130468000 -1.508736000

1 4.240347000 -0.001997000 -1.104104000

8 5.073816000 -1.126111000 0.275178000

1 5.919620000 -0.765195000 0.561125000

1 5.293723000 -1.905118000 -0.247763000

1 -1.201984000 3.385775000 -0.909378000

6 -0.425898000 2.760055000 -0.463077000

6 0.856060000 2.787988000 -0.951220000

6 1.595333000 1.220287000 0.544089000

6 0.318228000 1.122899000 1.161653000

6 -0.738903000 1.904514000 0.627024000

6 -2.035989000 1.778806000 1.190339000

6 -2.247643000 0.919636000 2.242009000

6 -1.186206000 0.148516000 2.778948000

6 0.077386000 0.239527000 2.247388000

1 1.120875000 3.440846000 -1.788208000

1 2.385619000 0.547871000 0.895998000

1 0.908892000 -0.372044000 2.609451000

1 -1.382901000 -0.531841000 3.610340000

1 -3.248484000 0.822173000 2.668760000

1 -2.858445000 2.365202000 0.774619000

1 -0.008739000 0.243660000 -2.831533000

6 -0.155370000 -0.454485000 -2.005359000

6 0.927259000 -1.063572000 -1.420758000

6 -0.381294000 -2.186579000 0.094192000

6 -1.575503000 -1.607206000 -0.412576000

6 -1.457018000 -0.700011000 -1.498357000

6 -2.630425000 -0.070275000 -1.995215000

6 -3.853030000 -0.340264000 -1.430789000

6 -3.968510000 -1.251388000 -0.349967000

6 -2.851461000 -1.872232000 0.150142000

1 1.945389000 -0.820072000 -1.745056000

1 -0.443549000 -2.874848000 0.946173000

1 -2.926738000 -2.569761000 0.988033000

1 -4.950030000 -1.450249000 0.085093000

1 -4.750191000 0.148945000 -1.816836000

1 -2.542673000 0.634472000 -2.825392000

7 1.860121000 2.017593000 -0.461890000

7 0.813047000 -1.925213000 -0.374753000

E_SCF_ = -1032.08909 a.u.

**(Pyra)_2_**

7 1.001424000 -0.230830000 -1.440480000

6 1.279680000 0.999825000 -1.020017000

6 1.469370000 -1.229508000 -0.696088000

6 2.212910000 -0.997344000 0.462653000

1 0.890900000 1.836357000 -1.608905000

1 1.239432000 -2.250352000 -1.016224000

1 2.586332000 -1.833823000 1.062092000

7 -1.637035000 -1.385585000 0.452779000

6 -2.223234000 -0.858734000 -0.616813000

6 -1.155893000 -0.524673000 1.347482000

6 -1.265266000 0.855303000 1.172552000

1 -2.616521000 -1.549212000 -1.369508000

1 -0.663590000 -0.937382000 2.233726000

1 -0.860841000 1.546087000 1.919292000

7 -1.858689000 1.382972000 0.103442000

7 2.491425000 0.233662000 0.883685000

6 -2.335752000 0.523200000 -0.790351000

6 2.022994000 1.231519000 0.139652000

1 -2.820609000 0.935937000 -1.680655000

1 2.236174000 2.253326000 0.469777000

E_SCF_ = -528.0651844 a.u.

**Pyra_2_(H_2_O)**

7 1.885971000 1.199925000 -0.691059000

6 0.790034000 1.003922000 -1.419771000

6 1.738005000 1.857542000 0.456470000

6 0.488982000 2.315303000 0.878104000

1 0.902354000 0.466189000 -2.365635000

1 2.632129000 2.011328000 1.067586000

1 0.378710000 2.857173000 1.822182000

7 0.609444000 -2.091547000 -0.165652000

6 -0.491539000 -2.313815000 -0.878299000

6 0.465024000 -1.439067000 0.986898000

6 -0.787767000 -1.004283000 1.421250000

1 -0.383261000 -2.854895000 -1.823062000

1 1.383161000 -1.235917000 1.546345000

1 -0.898102000 -0.467329000 2.367793000

8 3.410625000 -1.199220000 0.163023000

1 3.191661000 -0.381582000 -0.307561000

1 2.759963000 -1.819966000 -0.193401000

8 -3.409896000 1.196357000 -0.165883000

1 -3.190552000 0.380668000 0.307913000

1 -2.760510000 1.819122000 0.189360000

7 -1.885126000 -1.199035000 0.694318000

7 -0.610581000 2.091909000 0.163603000

6 -1.739570000 -1.855791000 -0.454007000

6 -0.463780000 1.438459000 -0.988065000

1 -2.634865000 -2.008506000 -1.063670000

1 -1.380819000 1.234242000 -1.548935000

E_SCF_ = -680.7789585 a.u.

**Pyra_2_(H_2_O)_2_**

1 3.984319000 1.687009000 1.327647000

8 3.402778000 0.934737000 1.183708000

1 2.512480000 1.325566000 1.051052000

1 3.643441000 0.369128000 -0.600362000

8 3.507979000 0.116916000 -1.531330000

1 3.092218000 -0.754414000 -1.454060000

1 -3.079461000 0.346048000 -1.291818000

8 -3.593691000 -0.487366000 -1.303381000

1 -4.463559000 -0.248875000 -1.635626000

1 -3.613903000 -0.487253000 0.629814000

8 -3.371457000 -0.311793000 1.552928000

1 -2.516891000 -0.764379000 1.630588000

6 -0.333709000 1.941428000 1.071772000

6 -1.575507000 1.803235000 0.452103000

7 -1.661728000 1.500031000 -0.842638000

6 -0.523100000 1.333141000 -1.511946000

6 0.719645000 1.431134000 -0.886664000

7 0.802703000 1.736581000 0.410447000

1 -0.264840000 2.185316000 2.135393000

1 -2.503273000 1.858840000 1.024322000

1 -0.594115000 1.077370000 -2.572983000

1 1.657127000 1.195739000 -1.408353000

6 1.674583000 -1.629342000 0.766912000

6 0.532462000 -1.429627000 1.542653000

7 -0.688175000 -1.486144000 1.016311000

6 -0.777615000 -1.773361000 -0.282028000

6 0.366290000 -2.002185000 -1.049483000

7 1.588446000 -1.914095000 -0.531203000

1 2.670887000 -1.506103000 1.199111000

1 0.615954000 -1.193540000 2.607337000

1 -1.776268000 -1.777892000 -0.732178000

1 0.283995000 -2.233662000 -2.115625000

E_SCF_ = -833.5053594 a.u.

**Pyra_2_(H_2_O)_3_**

6 -1.418509000 2.101734000 -0.120197000

6 -0.199802000 2.210775000 -0.788842000

7 0.928231000 1.734152000 -0.267758000

6 0.853984000 1.165242000 0.936375000

6 -0.364557000 1.082070000 1.613389000

7 -1.497057000 1.541554000 1.085448000

1 -2.346636000 2.439246000 -0.585637000

1 -0.143635000 2.674053000 -1.778025000

1 1.764665000 0.718716000 1.358039000

1 -0.423019000 0.607067000 2.596860000

6 0.431855000 -1.197196000 -1.735916000

6 1.575695000 -1.548024000 -1.016322000

7 1.488916000 -2.074461000 0.203156000

6 0.266291000 -2.246316000 0.703746000

6 -0.875714000 -1.867501000 -0.001107000

7 -0.787194000 -1.346048000 -1.226989000

1 0.522422000 -0.753765000 -2.731292000

1 2.562193000 -1.313830000 -1.422773000

1 0.185011000 -2.669531000 1.709123000

1 -1.868987000 -1.931133000 0.455521000

1 2.863538000 -1.337406000 1.439417000

8 3.317318000 -0.650886000 1.957008000

1 4.009693000 -0.326222000 1.363408000

8 3.347034000 0.820318000 -1.853553000

1 4.040562000 0.801551000 -1.179651000

1 2.601028000 1.241760000 -1.396948000

8 5.323968000 0.558408000 0.254618000

1 5.648564000 1.349714000 0.697869000

1 6.111229000 0.055177000 0.020929000

1 -2.537952000 -0.504024000 -1.659445000

8 -3.300705000 -0.017848000 -1.300175000

1 -3.198503000 -0.180010000 -0.343644000

8 -3.437588000 -0.467185000 1.471878000

1 -4.366585000 -0.302060000 1.221236000

1 -3.018186000 0.406500000 1.570573000

8 -5.731784000 -0.088543000 -0.083622000

1 -5.079533000 -0.222198000 -0.798495000

1 -6.017564000 0.821208000 -0.210057000

E_SCF_ = -986.2132326 a.u.

**(Thia)_2_**

6 1.442214000 0.052133000 1.317228000

6 2.228053000 -0.424151000 -0.918038000

6 1.215767000 -1.214054000 0.857022000

1 1.173846000 0.480244000 2.280450000

1 2.654822000 -0.380816000 -1.921946000

1 0.709589000 -1.996987000 1.423028000

6 -2.285949000 0.898777000 0.669127000

6 -1.489077000 1.509641000 -0.257122000

6 -1.385428000 -0.523878000 -1.067686000

1 -2.792641000 1.342464000 1.523618000

1 -1.241231000 2.571909000 -0.257806000

1 -1.090499000 -1.359427000 -1.705564000

7 1.664487000 -1.475022000 -0.410074000

7 -0.985726000 0.696578000 -1.236436000

16 2.258758000 0.963944000 0.110493000

16 -2.416176000 -0.776387000 0.300794000

E_SCF_ = -1137.477976 a.u.

**Thia_2_(H_2_O)**

8 1.111278000 2.473686000 -0.057629000

1 0.333577000 2.138045000 -0.533115000

1 1.826293000 1.918221000 -0.385917000

6 -2.517413000 0.252026000 1.277199000

6 -2.364361000 0.552360000 -1.122564000

6 -1.406676000 0.902287000 0.829728000

1 -2.790589000 0.004871000 2.300809000

1 -2.519638000 0.555750000 -2.202838000

1 -0.591984000 1.290463000 1.441480000

6 3.321374000 -0.019163000 0.837011000

6 2.236872000 -0.582929000 1.440724000

6 1.574306000 -0.809536000 -0.647232000

1 4.207865000 0.410645000 1.298147000

1 2.108217000 -0.691529000 2.517551000

1 0.926049000 -1.091213000 -1.479948000

7 -1.337604000 1.072723000 -0.529715000

7 1.260663000 -1.031328000 0.591490000

16 -3.518237000 -0.175973000 -0.060084000

16 3.108599000 -0.049043000 -0.873265000

8 -1.013336000 -2.353783000 -0.457542000

1 -1.857185000 -1.949065000 -0.232938000

1 -0.397949000 -2.005187000 0.210100000

E_SCF_ = -1290.1825297 a.u.

**Thia_2_(H_2_O)_2_**

8 1.679217000 1.863213000 1.691089000

1 2.396204000 1.280993000 1.417769000

1 1.493193000 2.368095000 0.876578000

8 0.923026000 3.075641000 -0.731243000

1 0.783645000 4.022187000 -0.632062000

1 0.026160000 2.687967000 -0.659610000

6 -2.143637000 -0.159615000 1.145030000

6 -1.175553000 0.754523000 0.856247000

6 -2.719278000 1.471716000 -0.555305000

1 -2.087026000 -1.015256000 1.814850000

1 -0.183660000 0.824759000 1.314384000

1 -3.198466000 2.076898000 -1.326996000

6 2.135791000 0.163090000 -1.145657000

6 1.177409000 -0.759433000 -0.851064000

6 2.738125000 -1.468427000 0.545024000

1 2.066468000 1.021012000 -1.811391000

1 0.182668000 -0.835253000 -1.302029000

1 3.228829000 -2.071858000 1.310872000

7 1.532543000 -1.674063000 0.113110000

7 -1.515832000 1.669224000 -0.113358000

16 3.546360000 -0.137420000 -0.201661000

16 -3.543680000 0.149290000 0.188227000

8 -0.941652000 -3.086513000 0.745193000

1 -0.808286000 -4.034332000 0.649519000

1 -0.042566000 -2.706865000 0.680861000

1 -1.500036000 -2.375726000 -0.868337000

8 -1.685115000 -1.868260000 -1.681158000

1 -2.397937000 -1.282440000 -1.404421000

E_SCF_ = -1442.909012 a.u.

**Thia_2_(H_2_O)_3_**

8 3.254921000 -1.163763000 1.826813000

1 2.643688000 -1.603358000 1.205632000

1 3.954846000 -0.828055000 1.249622000

8 3.366150000 0.787011000 -1.918494000

1 2.819824000 1.313533000 -1.307020000

1 4.035716000 0.404430000 -1.334417000

8 5.302088000 -0.095619000 0.058941000

1 5.706455000 0.700217000 0.420669000

1 6.034114000 -0.687907000 -0.141435000

6 -0.344983000 -1.105660000 -1.409267000

6 0.967553000 -1.216456000 -1.060606000

6 0.118648000 -2.529707000 0.499431000

1 -0.818122000 -0.515651000 -2.190205000

1 1.796041000 -0.680176000 -1.536087000

1 0.078551000 -3.193904000 1.365165000

6 -0.172242000 1.214792000 1.415586000

6 1.138485000 1.161421000 1.043356000

6 0.420443000 2.547643000 -0.517381000

1 -0.688668000 0.698745000 2.221245000

1 1.900743000 0.526256000 1.507489000

1 0.441619000 3.200596000 -1.392300000

7 1.460639000 1.922750000 -0.056289000

7 1.214859000 -2.024617000 0.026229000

16 -1.039662000 2.254706000 0.352647000

16 -1.311645000 -2.056567000 -0.346928000

1 -2.636006000 0.822702000 -0.802290000

8 -3.052703000 0.300830000 -1.502431000

1 -3.927857000 0.109236000 -1.139388000

8 -5.466801000 -0.178239000 0.043123000

1 -5.961087000 -1.001388000 -0.029044000

1 -6.127838000 0.521702000 0.022639000

1 -3.908324000 -0.005835000 1.200404000

8 -2.990829000 0.088751000 1.491617000

1 -2.521323000 -0.582266000 0.974968000

E_SCF_ = -1595.6028294 a.u.

**(Oxa)_2_**

6 -1.557487000 1.101648000 -0.455480000

6 -1.464099000 -1.004537000 -0.545371000

6 -2.140284000 0.617496000 0.669804000

1 -1.397464000 2.135282000 -0.751962000

1 -1.308562000 -2.052466000 -0.793442000

1 -2.607279000 1.060508000 1.543379000

6 1.412935000 0.470152000 1.175614000

6 1.491788000 -0.849688000 0.868819000

6 1.964961000 0.322063000 -0.854078000

1 1.155311000 0.935472000 2.124260000

1 1.346680000 -1.775451000 1.416115000

1 2.229360000 0.475735000 -1.897688000

7 -1.130471000 0.038398000 -1.227299000

7 1.726081000 1.208852000 0.051270000

8 -2.084053000 -0.735780000 0.612792000

8 1.854778000 -0.945798000 -0.433332000

E_SCF_ = -491.621581 a.u.

**Oxa_2_(H_2_O)**

8 -2.893442000 1.693820000 -0.525871000

1 -2.358449000 1.756883000 0.275080000

1 -2.387688000 1.075909000 -1.063763000

6 1.639928000 1.658271000 -0.565643000

6 0.920996000 0.982437000 1.339518000

6 0.343185000 2.020738000 -0.412945000

1 2.386514000 1.772830000 -1.344694000

1 0.999851000 0.479569000 2.300544000

1 -0.314242000 2.560976000 -1.090528000

6 -2.069412000 -1.230563000 0.259652000

6 -1.186840000 -1.925493000 1.017352000

6 -0.179529000 -1.338071000 -0.751154000

1 -3.095842000 -0.896125000 0.370756000

1 -1.337084000 -2.384358000 1.991565000

1 0.538757000 -1.145396000 -1.546086000

7 -0.098712000 1.575904000 0.821240000

7 0.025126000 -1.979330000 0.351146000

8 2.013583000 0.996137000 0.560604000

8 -1.425231000 -0.859994000 -0.877062000

8 2.822841000 -1.614246000 -0.777665000

1 2.847641000 -0.871445000 -0.164231000

1 2.283673000 -2.254507000 -0.296077000

E_SCF_ = -644.3289701 a.u.

**Oxa_2_(H_2_O)_2_**

8 3.628908000 0.138036000 -0.564947000

1 3.020843000 0.888927000 -0.514347000

1 4.505596000 0.518080000 -0.454654000

8 2.506053000 -1.058698000 1.827483000

1 1.797384000 -1.569647000 1.386177000

1 3.072248000 -0.803983000 1.082799000

6 -0.871801000 1.947075000 -0.278876000

6 0.293932000 2.370584000 -0.820213000

6 0.714626000 1.197097000 0.940713000

1 -1.901143000 2.047081000 -0.622850000

1 0.567156000 2.968258000 -1.683396000

1 1.360965000 0.627405000 1.620328000

6 0.898639000 -1.429881000 -1.119814000

6 -0.796721000 -1.872848000 0.083547000

6 -0.205513000 -1.118794000 -1.839711000

1 1.950262000 -1.308957000 -1.373549000

1 -1.512952000 -2.089686000 0.874192000

1 -0.388086000 -0.719435000 -2.831679000

7 0.492976000 -1.915744000 0.113602000

7 -0.573837000 1.201979000 0.851503000

8 -1.290008000 -1.406437000 -1.074087000

8 1.308988000 1.893501000 -0.043198000

1 -1.915848000 0.236535000 1.677078000

8 -2.700885000 -0.327609000 1.857207000

1 -3.055487000 -0.016173000 2.694289000

1 -3.501440000 0.240793000 0.246715000

8 -3.538450000 0.463977000 -0.699967000

1 -2.949288000 -0.204390000 -1.070649000

E_SCF_ = -797.0535287 a.u.

**Oxa_2_(H_2_O)_3_**

8 -3.524635000 1.312251000 0.523886000

1 -2.887098000 1.584596000 -0.147672000

1 -4.033845000 0.601237000 0.108809000

8 -2.409433000 -1.923827000 -1.053414000

1 -1.787003000 -1.972554000 -0.316732000

1 -3.255566000 -1.691144000 -0.645203000

8 -5.000474000 -1.024704000 -0.214534000

1 -5.473200000 -1.295772000 0.579688000

1 -5.619565000 -1.150851000 -0.941395000

6 1.056384000 1.651029000 -0.877399000

6 -0.009416000 2.486324000 -0.878220000

6 -0.641611000 0.531012000 -1.513549000

1 2.078455000 1.814436000 -0.528462000

1 -0.165997000 3.530210000 -0.628282000

1 -1.368380000 -0.244658000 -1.770401000

6 0.176553000 0.603401000 2.218395000

6 -0.937651000 -0.001823000 1.742283000

6 0.788065000 -1.236645000 1.356830000

1 0.270168000 1.567855000 2.711062000

1 -1.982760000 0.288214000 1.630807000

1 1.321098000 -2.055882000 0.872603000

7 1.271890000 -0.202656000 1.960624000

7 0.624287000 0.401347000 -1.294885000

8 -0.544636000 -1.189285000 1.200504000

8 -1.098085000 1.773713000 -1.285103000

1 1.660064000 -1.325500000 -1.314699000

8 2.197045000 -2.117652000 -1.133421000

1 3.080719000 -1.751977000 -0.988169000

8 4.808413000 -0.881367000 -0.795649000

1 5.140315000 -0.574322000 -1.645931000

1 5.528149000 -1.388492000 -0.406050000

1 4.055753000 0.510449000 0.322233000

8 3.646872000 1.205530000 0.856648000

1 2.970969000 0.726245000 1.366256000

E_SCF_ = -949.752736 a.u.
